# Validation and Calibration of a Model Used to Reconstruct Historical Exposure to Polycyclic Aromatic Hydrocarbons for Use in Epidemiologic Studies

**DOI:** 10.1289/ehp.8659

**Published:** 2006-03-13

**Authors:** Jan Beyea, Maureen Hatch, Steven D. Stellman, Regina M. Santella, Susan L. Teitelbaum, Bogdan Prokopczyk, David Camann, Marilie D. Gammon

**Affiliations:** 1 Consulting in the Public Interest, Lambertville, New Jersey, USA; 2 Division of Cancer and Epidemiology and Genetics, National Cancer Institute, National Institutes of Health, Department of Health and Human Services, Rockville, Maryland, USA; 3 Department of Epidemiology and; 4 Department of Environmental Health Sciences, Mailman School of Public Health, Columbia University, New York, New York, USA; 5 Department of Community and Preventive Medicine, Mount Sinai School of Medicine, New York, New York, USA; 6 College of Medicine, Penn State University, Hershey, Pennsylvania, USA; 7 Southwest Research Institute, San Antonio, Texas, USA; 8 Department of Epidemiology, University of North Carolina at Chapel Hill, Chapel Hill, North Carolina, USA

**Keywords:** breast cancer, calibration, carpet dust, DNA adducts, PAH, soil, traffic, validation

## Abstract

**Objectives:**

We previously developed a historical reconstruction model to estimate exposure to airborne polycyclic aromatic hydrocarbons (PAHs) from traffic back to 1960 for use in case–control studies of breast cancer risk. Here we report the results of four exercises to validate and calibrate the model.

**Methods:**

Model predictions of benzo[*a*]pyrene (BaP) concentration in soil and carpet dust were tested against measurements collected at subjects’ homes at interview. In addition, predictions of air intake of BaP were compared with blood PAH–DNA adducts. These same soil, carpet, and blood measurements were used for model optimization. In a separate test of the meteorological dispersion part of the model, predictions of hourly concentrations of carbon monoxide from traffic were compared with data collected at a U.S. Environmental Protection Agency monitoring station.

**Results:**

The data for soil, PAH–DNA adducts, and carbon monoxide concentrations were all consistent with model predictions. The carpet dust data were inconsistent, suggesting possible spatial confounding with PAH-containing contamination tracked in from outdoors or unmodeled cooking sources. BaP was found proportional to other PAHs in our soil and dust data, making it reasonable to use BaP historical data as a surrogate for other PAHs. Road intersections contributed 40–80% of both total emissions and average exposures, suggesting that the repertoire of simple markers of exposure, such as traffic counts and/or distance to nearest road, needs to be expanded to include distance to nearest intersection.

Geographic modeling, using emissions data and transport models, strives to create the equivalent of a hypothetical, ideal monitoring system that would have measured the concentration of pollutants at all locations and times in the medium and domain under study (Beyea and Hatch 1999). Such models, which are becoming increasingly more common in environmental epidemiology (Nuckols et al. 2004), represent a relatively new method for moving beyond the ecological studies that have dominated past work. Once validated, these models can reduce exposure misclassification by allowing the assignment of individualized, rather than average, exposures to study subjects.

We have constructed a geographic model for airborne polycyclic aromatic hydrocarbons (PAHs) from traffic that is being used in a population-based, case–control epidemiologic study involving about 3,000 women on Long Island, New York, known as the Long Island Breast Cancer Study Project (LIBCSP; Gammon et al. 2002a). The study area and surrounding traffic network are shown in Figure 1. The model is also being used in a similar study in Buffalo, New York (Nie et al. 2005).

Just as with a real monitoring system, it is possible to both validate and calibrate a geographic model. For this purpose we used samples collected in the LIBCSP from subsets of subjects: *a*) soil PAHs at residence, *b*) carpet PAH, and *c*) PAH–DNA adducts assessed in peripheral blood. Details of the measurements have been reported previously (Gammon et al. 2002a; Shantakumar et al. 2005). Additional information is provided in the accompanying online Supplemental Material (http://www.ehponline.org/docs/2006/8659/suppl.pdf). Details of and default parameters for the geographic model are also available (Beyea et al. 2005; Beyea J, Hatch M, Stellman SD, Gammon MD, unpublished data). We refer to a model before calibration as a “default” model and a model after calibration as an “optimized” model.

In addition to the samples collected as part of the LIBCSP, data collected by the U.S. Environmental Protection Agency (EPA) on concentrations of carbon monoxide were used as a test of the basic meteorological dispersion component of the model. A comparison of the historical emissions data used in the model has been made to sediment PAH concentrations and air measurements; the results will be reported elsewhere (Beyea J, Hatch M, Stellman SD, Gammon MD, unpublished data).

## Materials and Methods

Individual exposure estimates were generated using a meteorological dispersion model (Beyea et al. 2005) applied to estimates of PAHs emitted along hundreds of thousands of street segments (in units of nanograms per kilometer). Emission data per street segment were derived from historical data obtained for tailpipe emissions and number of vehicles on roads. Receptor locations localized to the street level were obtained by geocoding residence addresses obtained at interview. The model has two distinct components related to the temperature of the engines of emitting vehicles. “Warm-engine” emissions occur throughout the traffic network, whereas “cold-engine” emissions occur only for a relatively short distance from the vehicle starting point (default value, 1 km). Cold-engine emissions differ from warm-engine emissions in magnitude, in geographic location, and by time of day. For both warm- and cold-engine conditions, emissions are restricted in the model to times when the vehicles are traveling on major roads.

Vehicle emissions of PAHs are known to vary based on acceleration/deceleration conditions and the engine temperature, although the magnitude of the intersection contribution has not been quantified. Intersection emissions were taken proportional to warm-engine or cold-engine emissions on a particular street but restricted to a parameterized intersection distance, initially 100 m. One proportionality factor was taken for all warm-engine emissions and one for cold-engine emissions. Emissions could be graded further within one-half and one-quarter of the intersection distance.

Total emissions were written as the sum of five terms: (warm-engine emissions) + *A* × (warm-engine intersection emissions) + *B* × (cold-engine emissions) + *C* × (cold-engine intersection emissions) + *D* × background. The parameters *A*, *B*, *C*, and *D*, which are defined relative to the first term in the summation, were determined from fits to either the soil or DNA adduct data that minimized chi squared (Press et al. 1992). This chi-square minimization process was carried out while simultaneously varying, and thereby optimizing, a range of other model parameters such as washout rate, particle deposition rates, photo decay rates, and intersection distance.

### Validation data

#### PAH soil data

Soil measurements were chosen as a potential validation and calibration opportunity for the geographic model because deposition of PAHs is proportional to airborne concentrations above the soil (Odabasi et al. 1999) and because respirable particles in outdoor air are known to penetrate indoors efficiently and have been found to dominate indoor respirable PAH concentrations in a number of studies (Dubowsky et al. 1999; Sheldon et al. 1992, 1993). Soil measurements are easier to make than air measurements and retain historical information (Jones 1991).

#### PAH–DNA adducts

Airborne PAHs can enter the blood through the respiratory pathway, where they can be metabolized and form PAH–DNA adducts. If our model is valid, its predictions of recent airborne concentrations should be correlated with PAH–DNA adduct levels in study subjects, provided the traffic contribution is large enough to be detected. Previous studies have demonstrated that DNA adduct levels in white blood cells reflect short-term environmental exposure, if exposures are high enough (Eder 1999). For example, in a study by Binkova et al. (1995), a scattergram of exposure accumulated on personal dosimeters versus adduct levels showed a clear trend with only 21 subjects. Although the ambient concentrations were perhaps 10-fold higher than current U.S. levels, ranging from 1.6 to 2.9 ng/m^3^ of benzo[*a*]pyrene (BaP), we have the advantage of being able to work with many more subjects. We measured PAH–DNA adducts in the peripheral blood of 999 study subjects, using the competitive ELISA method, as described by Gammon et al. (2002b).

About 72% of women had detectable levels of adducts. We analyzed detects and non-detects separately because descriptive statistics indicated the existence of a bimodal distribution for adduct level. The data show a normal distribution with a large spike at the origin that is well separated from, and not part of, the normal distribution. The nondetect spike contains 28% of the women in the sample.

Previous work with this study population has found that the likelihood of having detectable adducts is elevated among past and current smokers, inversely associated with increased BaP levels (nanograms per gram) in dust in the home but positively associated with BaP levels in soil outside of the house, although confidence intervals were large (Shantakumar et al. 2005). The study authors did not find any consistent associations between the odds of having detectable PAH–DNA adducts and various dietary sources of PAH, including smoked and grilled foods eaten in the most recent decade of life and a BaP food index assessed from responses to a food frequency questionnaire (Shantakumar et al. 2005). As suggested previously (Dickey et al. 1997), persons with and without detectable adduct levels may represent two different groups of individuals in their response to PAH exposure. Two distinct populations responding to PAHs in diet have also been reported (Kang et al. 1995). The first group of women, those with detectable adducts, are the focus of this report. It is easier to model the number of adducts in detects as a function of airborne PAH exposure than to predict the shift from nondetects to detects. The model reported in this article is not able to predict the odds ratio of having detectable adducts (Shantakumar et al. 2005). Regardless of whether the distribution in adduct levels reflects a bimodal biologic response or a bimodal exposure distribution, levels of DNA adducts reflect DNA damage and therefore serve as a measure of the effective biologic dose of PAHs (Binkova et al. 1995; Nesnow et al. 1993).

#### PAHs in carpet dust

PAHs in carpet dust come from three sources: ambient outdoor air PAHs that penetrate indoors and deposit on carpet, indoor-generated PAHs, and dirt-containing PAHs that are tracked from the outside. The geographic model should be able to predict the variation in the amount of ambient PAH deposition per square meter for wall-to-wall carpeting. For rugs and other carpet that do not extend to the walls, an ambiguity arises about what denominator to use, for example, carpet area or floor area because carpet may act as a “sink” for household dust deposited on the uncarpeted floor. Nevertheless, unless there is strong spatial confounding with carpet size, indoor-generated sources of PAHs (e.g., cooking), and/or dust track-in, we expect that the ambient signal should be detectable in the carpet PAH measurements that were collected as part of the LIBCSP to provide an exposure marker inclusive of indoor-generated PAHs.

#### CO air concentrations

Modeling CO air concentration offers a good test of a PAH dispersion and traffic model for a number of reasons. First, traffic is known to dominate CO emissions, accounting for as much as 95% of emissions in cities (U.S. EPA 2001). Therefore, both CO and traffic PAH emissions will increase and decrease with traffic density. Second, CO and PAHs are both associated with incomplete combustion (An and Ross 1996; Bostrom et al. 2002), so relative CO emissions should rise and fall during the driving cycle in a pattern that is similar to that of PAH. [As referenced in the Supplemental Material (http://www.ehponline.org/docs/2006/8659/suppl.pdf), hourly patterns of PAH and CO air concentrations have been found in other studies to be similar indoors and out, with *R*^2^ coefficients ranging from 0.5 to 0.8.] Third, to model relative hourly CO air concentrations in any single year, all the modeler has to do is turn off all depletion phenomena, because deposition, washout, and photo decay are negligible in the case of CO.

CO data are widely available for locations around the United States through requests to the U.S. EPA. In our study area, hourly CO data have been collected since 1974 at Eisenhower Park in Nassau County (U.S. EPA 2005). We averaged hourly data for 1975, 1985, and 1995 and regressed the results against comparable model predictions.

### Statistical methods

Multiple (linear) regression was used to assess and optimize the relationship between model predictions and CO data, as well as to compare adduct data with soil and dust data. In model fitting to soil, dust, and detectable adduct levels, the variables and the model predictions were all log-transformed to bring them to normal form. Because the model parameters to be determined appeared inside the transforming logarithm, nonlinear regression was required. When covariates, such as cooking sources or BaP in diet, were controlled for, a combination of nonlinear and multiple regression methods was used as discussed in the Supplemental Material (http://www.ehponline.org/docs/2006/8659/suppl.pdf). Fits to the adduct and dust data were made with and without current smokers included.

Significance values for bimodal distributions of PAH–DNA adducts were handled using the method of Simes and Hochberg, as described by Levin (1996). According to this method, to obtain an overall significance of 95%, a *p*-value < 0.025 is required for one of the modes whenever a *p*-value for the other mode is > 0.05 (Levin 1996). Because all *p*-values for adduct nondetects in this study are greater than 0.05, we must always look for a *p*-value ≤ 0.025, for correlations using detectable adduct values alone.

## Results

### Model validation and calibration

#### CO data

When normalized, hourly CO data in 1975 were virtually identical to the hourly data in 1995, despite a 4-fold drop in absolute levels. Because model regressions for 1975, 1985, and 1995 were similar, we discuss only the 1995 results.

With the default model parameter values chosen before optimization, there is a reasonable fit to the 1995 hourly CO data for the Nassau County monitor (*r*^2^ = 52%). This rough agreement is no trivial result because the *r*^2^ for the correlation between hourly CO emissions and concentrations was only 0.033. This weak correlation arises because CO emissions are very low in the early morning hours in contrast to the measured CO concentrations, which are relatively high at this time, reaching half the maximum daytime value. The delay time involved in distant CO reaching a receptor explains the result. CO measured at 0200 hr was actually emitted miles away during the tail of the evening rush hour.

**Figure 2:**
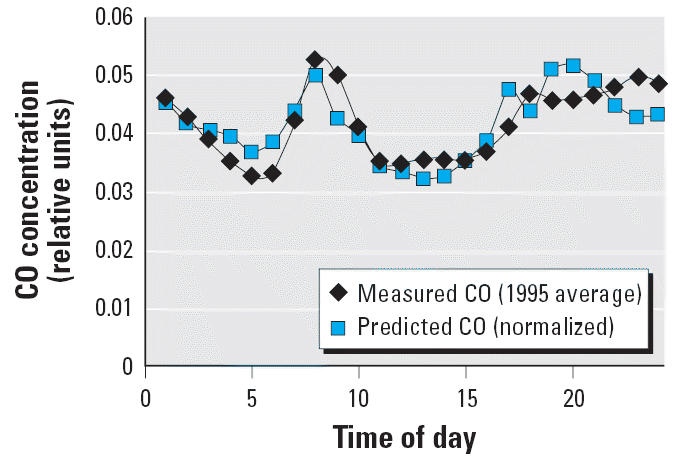
Relative CO hourly data: model predictions after optimization versus measurements averaged over 1 year.

Although the default model accounts for delay in CO arrival, the default model over predicts in the early evening hours. The over prediction remains despite the type of dispersion parameters used (urban or rural), despite a switch to a different year’s meteorological data, and despite the method chosen to convert the actual dispersion values from raw meteorological data. However, optimization of the fit to the CO data, allowing the relative strength of the intersection emissions and the contribution from distant sources (background term) to increase over the default value, eliminated the overprediction. The model parameters determined from the CO optimization were qualitatively similar to those determined from the soil data but differed from the parameters determined with PAH–DNA adduct data. The results of the fit to the CO data after optimization (*r*^2^ = 63%) are shown in Figure 2.

The fact that the optimized CO regression results for 1975 and 1985 were similar to the 1995 results suggests that emissions at intersections have been dominant over the entire period for which data are available.

#### Soil data

There was a high degree of correlation between BaP data and other PAHs [Supplemental Material (http://www.ehponline.org/docs/2006/8659/suppl.pdf)]. The mean soil level was 2,300 ng/g of BaP, which is approximately twice that reported in the only comparable data set we could find in the Northeast, namely, average values for Boston, Providence, and Springfield, Massachusetts, as reported by Bradley et al. (1994). The difference in mean soil levels may be attributable to differences in traffic density or differences in the depth of the samples collected in the two locations. The depth in the New England study ranged from 0 to 10 cm rather than the average 2-cm depth used in our study.

#### Spatial variation of the soil data

**Figure 3:**
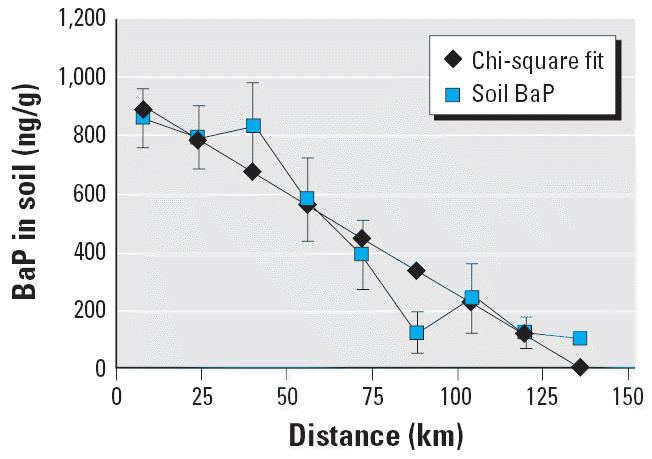
Soil PAHs (geometric mean in 16-km zones) as function of distance from urbanization along the length of Long Island. Error bars indicate SE.

**Figure 4:**
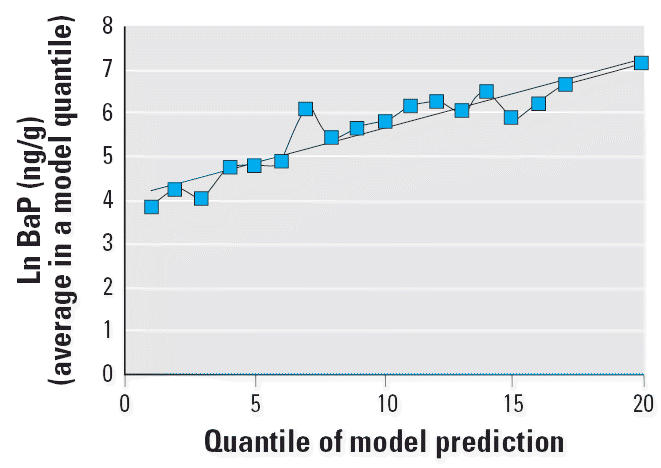
Average soil PAHs versus prediction of warm-engine model, by quantile. *r*^2^ = 0.8636.

Aggregated soil levels within geographic zones were found to decrease with distance along the axis of Long Island away from urbanization (and hence from pollution sources), as shown in Table 1 and Figure 3. The 10-fold decline in geometric mean values is consistent with other studies (Grass et al. 2000; Wagrowski and Hites 1997). Although there is considerable individual variation between prediction and soil data, the fit is highly significant [*p* < 0.0001, as discussed in the Supplemental Material (http://www.ehponline.org/docs/2006/8659/suppl.pdf)]. The fit to the soil data when the model predictions are grouped into 20 quantiles is shown in Figure 4. Further details about the soil regressions are given in the Supplemental Material (http://www.ehponline.org/docs/2006/8659/suppl.pdf) along with values for the correlation coefficients between aggregated soil, dust, and adduct data. In addition to allowing us to calibrate the model, the usefulness of the results obtained from the soil fits demonstrates the feasibility of using interviewer-phlebotomists on a tight schedule to gather environmental samples in the context of a large-scale case–control study.

The optimized parameter set determined from the soil data is shown in Table 2. Intersection emissions contribute 80% of average emissions and exposures when calculated with the optimized model.

#### Adduct data

**Figure 5:**
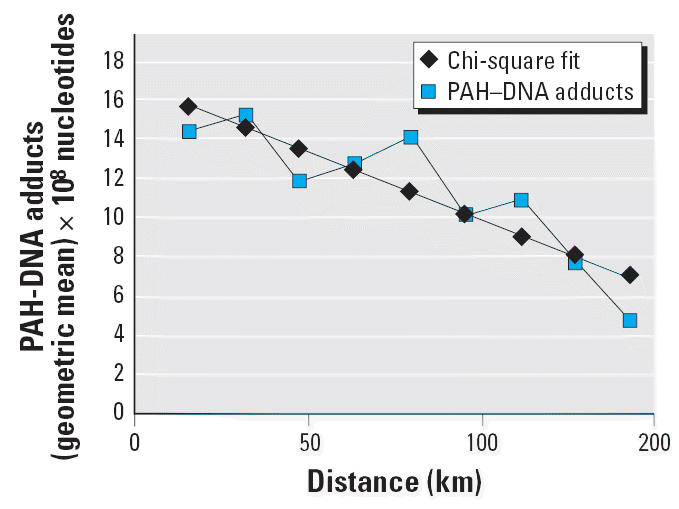
PAH–DNA adducts (geometric mean in 16-km zones) versus distance along the length of Long Island away from urbanization.

Aggregated adduct levels within geographic zones were found to decrease with distance along the axis of Long Island away from urbanization (and hence from pollution sources), as shown in Table 1 and Figure 5. The number of detects and nondetects for PAH–DNA adducts varies with 16-km zone along the length of Long Island [Supplemental Material, Table S-1 (http://www.ehponline.org/docs/2006/8659/suppl.pdf)]. The odds ratio of having detectable adducts does not differ significantly by zone (*p* = 0.23).

The results of optimization of the model to the DNA adduct data are shown in Table 2. Optimization produced a large coefficient for the cold-engine component, with the coefficient for warm emissions negligible. This is the reverse of the results for the soil-optimized model. On the other hand, when it came to the importance of intersection emissions, the results of fits to the adduct data were consistent with the fits to the soil data in predicting a major role for enhanced emissions at intersections. Intersections accounted on average for 40% of total cold-engine exposures. Deposition velocity, rain washout rate, and photo decay rate were all optimized at zero, which meant that the optimized adduct model did not contain any depletion. The parameter values were not changed significantly upon removing from the regressions women who smoked within the last year, nor were they changed significantly when we controlled for a BaP food index, assessed from responses to a food frequency questionnaire, and the number of smoked and grilled foods eaten in the most recent decade of life, also obtained from a questionnaire.

**Figure 6:**
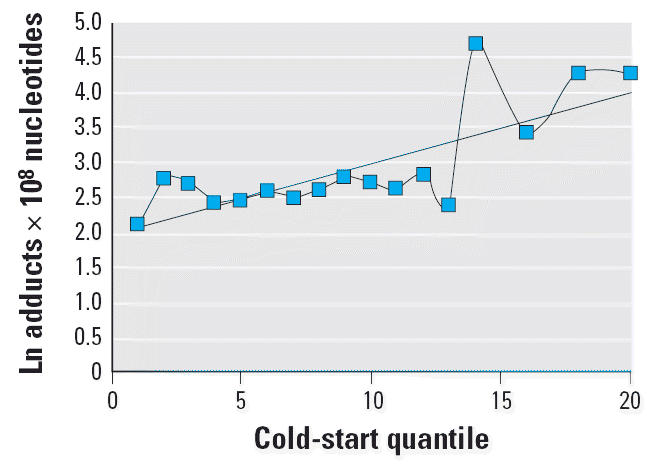
Average PAH–DNA adducts by exposure quantile predicted by cold-engine model. *r*^2^ = 0.58.

The fit of the adduct-optimized model to the adduct data is shown in Figure 6.The fit to the ungrouped data points can be found in the Supplemental Material (http://www.ehponline.org/docs/2006/8659/suppl.pdf; *p* = 0.02 before optimization). The results are not as good as in the soil case, with the cold-engine version tracking the adduct data less well than the warm-engine version tracks the soil data. The *r*^2^ for the grouped data is lower, at 58%.

Of interest is the fact that the soil data were comparable with the geographic model in predicting PAH–DNA adduct levels in individual women with detectable adducts (*p* = 0.004). Although soil data appear to be a simpler indicator of airborne exposure than the geographic model, soil data are not available for all women, nor are they available historically.

#### Dust data

Values for both PAHs per gram of dust and PAHs per square meter of carpet vacuumed were available. The Pearson correlation coefficients between BaP and the other two PAHs measured, dibenz[*a*,*h*]anthracene and benz[*a*]anthracene, were > 0.95. The trend in carpet BaP/m^2^ shows a peak in the center of Long Island (Table 1), indicating that some source of PAHs other than outside air is dominating BaP in carpets. Differences by zone were statistically significant. The same pattern is seen for BaP/g. In contrast, the total grams of dust per square meter behaves as expected, with high values closer to the urbanized portion of Long Island [Supplemental Material, Tables S-2, S-3 (http://www.ehponline.org/docs/2006/8659/suppl.pdf)]. Possible candidates for nontraffic PAHs are indoor sources such as cooking, and track-in of PAHs from outdoors. This is the one validation exercise that contradicts the model.

We found no explanation for this unexpected behavior of carpet dust with distance. Also anomalous was the correlation between BaP per square meter and the model’s prediction of deposition of ambient BaP onto carpet. In fact, the regression slope was negative and remained so even when potential confounders were included in the regression. Potential confounders considered were years in residence, work status, age, use of wood in stove/fireplace, number of children younger than 20 years at time of data collection, season, number of adults in the home, number of hours worked away from home, religion, education, income, smoking status, and the number of times a study subject consumed grilled meat or fish in the previous decade. This latter variable was the most relevant surrogate we had for cooking intensity. Excluding homes of study subjects who smoked within the last year did not reverse the negative correlation.

At the individual level, BaP in carpet, whether measured in units of nanograms per gram or nanograms per square meter, was not correlated with the level of measurable PAH–DNA adducts in study subjects (*p* > 0.46). As previously reported, a negative correlation between nanograms per gram of BaP in carpet dust was found in this population for the odds ratio of a woman having detectable adducts (Shantakumar et al. 2005). Clearly, much remains to be learned about the origins of PAHs in carpet dust.

The fact that the model correlated with the number of PAH–DNA adducts in women with detectable adducts, whereas the carpet dust data did not, suggests that the cause of the discrepancy with dust is unlikely to be connected with indoor sources of PAHs in the respirable range. Nevertheless, regardless of the size distribution, cooking is a likely source of PAHs in carpet dust that might be confounding the correlation with the geographic model. We only have a limited surrogate to use in controlling for cooking PAH.

Another potential contributor is track-in of PAHs from outdoors, which is a sequential process progressing from street, driveway, or attached garage to entryway and then to carpet. Such a pathway could include contamination at various points from vehicle oil drips, which could contribute track-in of PAHs distinct from blown soil dust.

Track-in can be a significant source of PAHs in carpet dust (Chuang et al. 1995). However, we have no explanation of why track-in patterns would vary spatially with distance along Long Island according to the pattern we found. It is thus not possible to rule out the possibility that some source of indoor PAH, such as cooking, is overwhelming any traffic contribution in carpets, particularly if the particle sizes are outside the respirable range.

#### Background model

Two background models were tested. Compared with a constant background term, a better fit to the data was found in calibrations using the soil data for a term that was proportional to exposures calculated from the more distant counties (all but Nassau, Suffolk, and Queens counties in New York State). The fit to the DNA adduct data was best with a constant background term.

## Discussion

The optimized model parameters can be compared with default values. For the fits to the soil data, the optimized parameters are within a factor of 2 of the default values for those dispersion parameters that are widely reported in the literature, namely, deposition velocity and washout rate (National Council on Radiation Protection and Measurement 1993; Ramsdell et al. 1994). For those parameters for which no strong guidance as to default values was available in the literature, for example, photo decay rates and acceleration/deceleration distances, the optimized values turned out to differ by more than a factor of 2 from the values we chose as defaults.

The most striking result to come out of the fits to the adduct data is the removal of depletion phenomena from the optimized cold-engine version of the model, whether it be dry deposition, wet deposition, or photo decay of PAHs. This artificial result is an indication that to optimize the fit, the model needs contributions from more distant sources than would normally be expected. Perhaps the best explanation is that, unlike soil receptors, human receptors are mobile. Or, perhaps indoor sources such as emissions of cooking PAHs for which we have not controlled, are confounding the results. Despite the apparent differences in the parameter values determined for the soil- and adduct-optimized models, the correlation between their predictions of PAH exposure for women in the study is quite high (*r*^2^ = 0.79–0.86), possibly because the difference in optimized parameters compensates for differences in spatial patterns that would otherwise result.

## Conclusion

This study indicates that in developing inhalation exposure estimates it is necessary to account for emissions at intersections to fully determine the spatial distribution of PAH exposure. Three of four validation exercises were consistent with model predictions. The unexpected geographic pattern of carpet PAHs, which does not match the falloff with distance from urbanization predicted by the geographic model, is the only result we found in our validation exercises that calls into question the relevance of our model. In contrast, the model predictions for soil PAH data and hourly CO concentrations were very consistent with the data, favoring a warm-engine emission model. PAH adduct levels for women with detectable adducts were also consistent with model predictions and favored a cold-engine emission model. Although we found a high degree of correlation between the predictions of the warm-engine version of the model and the cold-engine version, it will be prudent to use both the warm-engine and cold-engine versions when evaluating the effects of exposure on health outcomes.

Whatever model is used, the ability to make individualized exposure estimates has the potential to reduce exposure misclassification that can arise in environmental epidemiology studies from assigning group level exposure based on interpolation from sparse environmental monitoring data or from surrogate measures of exposure based on simple distance from nearest major road and/or traffic density. Although geographic models are complex, comparison of their output with field data helps to build confidence in them.

## Figures and Tables

**Figure 1:**
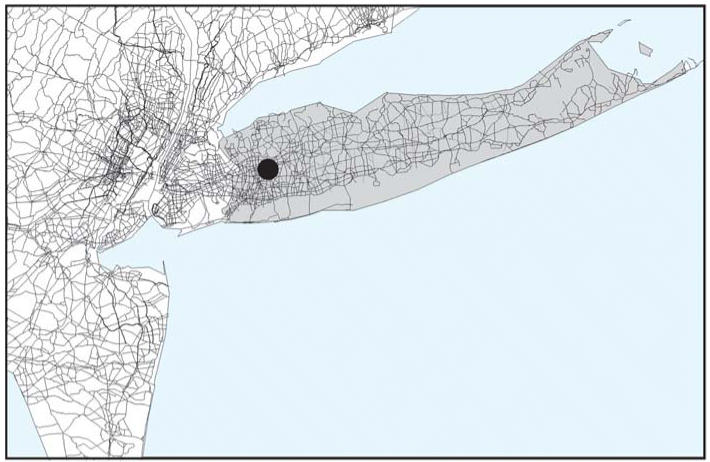
LIBCSP study area showing the major roads within an 80-km distance of Long Island from which vehicle emissions were tracked in this study. Study participants were drawn from the shaded area, which is 150-km in length and extends outward from New York City. The location of the U.S. Environmental Protection Agency carbon monoxide monitor is also indicated.

**Table 1:** Concentrations of PAH–DNA adducts, soil BaP, and BaP in carpet dust by 16-km geographic zones running from the most urbanized to the most rural end of Long Island.

	Adducts			Soil BaP					Carpet BaP		
Zone number^a^	Numbers (geometric mean)^b^	SE	Data points per zone^c^	Geometric mean (ng/g)^d^	SE	Arithmetic mean (ng/g)^d^	SE	Data points per zone^c^	Geometric mean (ng/m^2^)^e^	SE	Data points per zone^c^
1	14.4	1.15	136	860	100	2,100	350	132	570	81	151
2	15.2	1.04	137	790	110	2,500	460	140	870	130	162
3	11.8	1.21	88	830	150	2,800	520	100	1,400	270	98
4	12.7	1.29	68	580	140	2,600	590	77	1,560	350	70
5	14.0	2.40	30	390	120	1,000	280	30	980	420	28
6	10.2	1.79	10	120	70	220	110	7	490	470	9
7	10.9	5.17	8	240	120	430	200	8	1,080	870	6
8	7.6	4.03	6	120	54	160	62	5	310	130	4
9	4.7	5.51	2	100	54^f^	100	62^f^	1	NA	NA	0

NA, not applicable.

a Zones of 16 km measured from the Nassau County border eastward along the Long Island axis.

b Per 10^8^ nucleotides; includes only women with detectable adducts and a single residential address; seasonally adjusted.

c Data are included only for residences that could be geocoded to the street level. Study subjects selected for environmental sampling (soil and carpet) were required to have lived at their residence for ≥ 15 years. The number of sample points decreases rapidly with distance, consistent with the density of the population on Long Island, specifically the population of long-term residents.

d To a 2-cm depth. Values are standardized to date of collection. Mean of all data = 2,300. GM of all data = 700.

e The arithmetic mean data are quite similar.

f Taken equal to the value for zone 8.

**Table 2:** Optimized versus default parameters for exposure model.

	Parameter value		
Parameter^a^	Default	Warm-engine version (soil optimized)	Cold-start version (adduct optimized)
Deposition velocity (m/sec)	0.003	0.007^b^	0^b^
Washout rate	1^c^	One-half default	0
Airborne photo decay rate	0.01^d^	One-fourth default	0
Intersection contribution to average exposure (% of total)^e^	15%	80%	40%
Background (% of average exposure)^f^	10%	Five times default value	65%^g^
Cold-start length^h^	1 km	NA	0.5 km
Intersection distance^i^	100 m	12.5 m	500 m

NA, not applicable.

a Individual values of parameters are poorly determined, if taken out of the context of group-optimized values.

b Optimized value for fits to bootstrap samples can range from 0 to 0.03, but see footnote *a*.

c Multiplied by 1.42 × (precipitation rate in mm/hr)^0.75^ to give exponential decay rate in units of hr^−1^; from (Ramsdell et al. 1994), with typo corrected.

d Multiplied by pyranometer reading in Langleys per hour to give exponential decay rate in units of hr^−1^.

e Percentage contributions differ slightly for average emissions, particularly for the cold-start model.

f Background for the default and soil-optimized models varies spatially. It is taken proportional to the exposure generated by emissions in counties outside three counties that include or bound the study area (Nassau, Suffolk, and Queens counties). This spatially varying background model gave a better fit to the soil data than did a constant term.

g Constant background term.

h Distance from center of census block that vehicles emit at the cold-engine rate.

i Distance from intersection that vehicles emit at the acceleration/deceleration rate.

## References

[b1-ehp0114-001053] An F, Ross M (1996). A simple physical model for high power enrichment emissions. J Air Waste Manage Assoc.

[b2-ehp0114-001053] Beyea J, Hatch M (1999). Geographic exposure modeling: a valuable extension of GIS for use in environmental
epidemiology. Environ Health Perspect.

[b3-ehp0114-001053] Beyea J Hatch M Stellman SD Teitelbaum SL Gammon MD 2005. Development of a Traffic Model for Predicting Airborne PAH Exposures since 1960 on Long Island, New York. Report to the National Cancer Institute and the National Institute of Environmental Health Sciences for work completed under USPHS Grant U01-CA/ES-66572. Lambertville, NJ:Consulting in the Public Interest. Available: http://www.cipi.com/PDF/beyea2005trafficpahmodel.pdf [accessed 1 July 2005].

[b4-ehp0114-001053] Binkova B, Lewtas J, Miskova I, Lenicek J, Sram R (1995). DNA adducts and personal air monitoring of carcinogenic polycyclic aromatic
hydrocarbons in an environmentally exposed population. Carcinogenesis.

[b5-ehp0114-001053] Bostrom CE, Gerde P, Hanberg A, Jernstrom B, Johansson C, Kyrklund T (2002). Cancer risk assessment, indicators, and guidelines for polycyclic aromatic
hydrocarbons in the ambient air. Environ Health Perspect.

[b6-ehp0114-001053] Bradley LJN, Magee BH, Allen SL (1994). Background levels of polycyclic aromatic hydrocarbons (PAH) and selected
metals in New England urban soils. J Soil Contam.

[b7-ehp0114-001053] Chuang J, Callahan P, Menton R, Gordon S, Lewis R, Wilson N (1995). Monitoring methods of PAHs and their distribution in house dust and track-in
soil. Environ Sci Technol.

[b8-ehp0114-001053] Dickey C, Santella RM, Hattis D, Tang D, Hsu Y, Cooper T (1997). Variability in PAH-DNA adduct measurements in peripheral mononuclear cells: implications
for quantitative cancer risk assessment. Risk Anal.

[b9-ehp0114-001053] Dubowsky SD, Wallace LA, Buckley TJ (1999). The contribution of traffic to indoor concentrations of polycyclic aromatic
hydrocarbons. J Expo Anal Environ Epidemiol.

[b10-ehp0114-001053] Eder E (1999). Intraindividual variations of DNA adduct levels in humans. Mutat Res.

[b11-ehp0114-001053] Gammon MD, Neugut AI, Santella RM, Teitelbaum SL, Britton JA, Terry MB (2002a). The Long Island Breast Cancer Study Project: description of a multi-institutional
collaboration to identify environmental risk factors for breast
cancer. Breast Cancer Res Treat.

[b12-ehp0114-001053] Gammon MD, Santella RM, Neugut AI, Eng SM, Teitelbaum SL, Andrea Paykin A (2002b). Environmental toxins and breast cancer on Long Island. I. Polycyclic aromatic
hydrocarbon (PAH)-DNA adducts. Cancer Epidemiol Biomarkers Prev.

[b13-ehp0114-001053] Grass B, Hunter C, Theyverse S (2000). Gehalte an polycyclischen aromatischen Kohlenwasserstoffen (PAK) in Oberboeden
Hamburgs. Umweltwiss Schadstoff Forsch.

[b14-ehp0114-001053] Jones KC (1991). Contaminant trends in soils and crops. Environ Pollut.

[b15-ehp0114-001053] Kang DH, Rothman N, Poirier MC, Greenberg A, Hsu CH, Schwartz BS (1995). Interindividual differences in the concentration of 1-hydroxypyrene-glucuronide
in urine and polycyclic aromatic hydrocarbon-DNA adducts in peripheral
white blood cells after charbroiled beef consumption. Carcinogenesis.

[b16-ehp0114-001053] Levin B (1996). On the Holms, Simes, and Hochberg multiple test procedures. AmJ Public Health.

[b17-ehp0114-001053] National Council on Radiation Protection and Measurement 1993. Uncertainty in NCRP Screening Models Relating to Atmospheric Transport, Deposition and Uptake by Humans. Bethesda, MD:NCRP.

[b18-ehp0114-001053] Nesnow S, Ross J, Nelson G, Holden K, Erexson G, Kligerman A (1993). Quantitative and temporal relationships between DNA adduct formation in
target and surrogate tissues: implications for biomonitoring. Environ Health Perspect.

[b19-ehp0114-001053] Nie J, Beyea J, Bonner MR, Han D, Vena JE, Rogerson P (2005). Environmental exposure to traffic polycyclic aromatic hydrocarbons (PAHs) and
risk of breast cancer [Abstract]. Abstract 2183. Am Assoc Cancer Res.

[b20-ehp0114-001053] Nuckols JR, Ward MH, Jarup L (2004). Using geographic information systems for exposure assessment in environmental
epidemiology studies. Environ Health Perspect.

[b21-ehp0114-001053] Odabasi M, Sofuoglu A, Vardar N, Tasdemir Y, Holsen TM (1999). Measurement of dry deposition and air-water exchange of polycyclic aromatic
hydrocarbons with the water surface sampler. Environ Sci Technol.

[b22-ehp0114-001053] Press WH Teukolsky SA Vetterling WT Flannery BP 1992. Numerical Recipes in Fortran 77. Cambridge, UK:Cambridge University Press.

[b23-ehp0114-001053] Ramsdell JV Jr Simonen CA Burk KW 1994. Regional Atmosphere Transport Code for Hanford Emission Tracking (RATCHET). PNWD-2224 UC-000 HEDR. Richland, WA: Battelle Pacific Northwest Laboratories.

[b24-ehp0114-001053] Shantakumar S, Gammon MD, Eng SM, Sagiv SK, Gaudet MM, Teitelbaum SL (2005). Residential environmental exposures and other characteristics associated
with detectable PAH-DNA adducts in peripheral mononuclear cells in a
population-based sample of adult females.. J Expo Anal Environ Epidemiol.

[b25-ehp0114-001053] Sheldon L Clayton A Keever J Perritt R Whitaker D 1992. PTEAM: Monitoring of Phthalates and PAHs in Indoor and Outdoor Air Samples in Riverside California. Final Report, Vol 2. Sacramento, CA:California Environmental Protection Agency Air Resources Board, A933–A144.

[b26-ehp0114-001053] Sheldon L Whitaker D Keever J Clayton A Perritt R 1993. Phthalates and PAHs in indoor and outdoor air in a southern California community. In: Indoor Air ‘93: Proceedings of the 6th International Conference on Indoor Air Quality and Climate. Vol 3. Helsinki:Helsinki University of Technology, 109–114.

[b27-ehp0114-001053] U.S. EPA 2001. Latest Findings on National Air Quality: 2001 Status and Trends. Washington, DC:U.S. Environmental Protection Agency, Office of Air Quality Planning and Standards. Available: http://www.epa.gov/airtrends/aqtrnd01/carbon.html [accessed 25 February 2005].

[b28-ehp0114-001053] U.S. EPA 2005. AQS—Air Quality Subsystem. U.S. Environmental Protection Agency. Available: http://www.epa.gov/airs/aqs.html [accessed 7 March 2005].

[b29-ehp0114-001053] Wagrowski DM, Hites RA (1997). Polycyclic aromatic hydrocarbon accumulation in urban, suburban, and rural
vegetation. Environ Sci Technol.

