# Supplementary material for: Validation and Calibration of a Model Used to Reconstruct Historical Exposure to Polycyclic Aromatic Hydrocarbons for Use in Epidemiologic Studies
Source: Environ Health Perspect. 2006 Mar 13;114(7):1053–8. doi: 10.1289/ehp.8659 (PMC1513337; doi:10.1289/ehp.8659)

Supplement to:

Validation and calibration of a model used to reconstruct historical exposure to polycyclic aromatic hydrocarbons for use in epidemiologic studies

Jan Beyea, Maureen Hatch, Steven D. Stellman, Regina M. Santella, Susan L. Teitelbaum, Bogdan Prokopczyk, David Camann, and Marilie D. Gammon.

In this work, we used a modeling procedure (Beyea et al. 2005) that builds on a variety of traffic-related parameters, including tailpipe emissions and vehicle counts, for which substantial data resources exist over a long time period. The study area and surrounding traffic network are shown in Figure 1 of the main paper. We chose to model airborne exposure of PAH because studies to date have indicated that environmental exposure to airborne PAH can have a larger effect on blood PAH adduct levels than industrial exposure, food or smoking (Eder 1999).

Within the category of PAH air exposure, we focused on traffic emissions because they are a major source of both indoor and outdoor exposures to PAH, and often the largest source in areas near cities, as has been confirmed in a number of experimental studies ((Dubowsky et al. 1999), (Lim et al. 1999), (Harkov et al. 1984), (Dickhut et al. 2000)), (Dunbar et al. 2001) (Dunbar et al. 2001), (Levy et al. 2001). Traffic-related Benzo(a)pyrene (BaP) and other 4- to 6-ringed PAH compounds are found predominantly on sub-micron particles (Zielinska) that penetrate deep into the lung. An earlier case-control study of breast cancer on Long Island reported an elevated (but not statistically significant) odds ratio for breast cancer in areas of high traffic density (Lewis-Michl et al. 1996).

PAH vehicle emissions are known to vary based on acceleration/deceleration conditions and the engine temperature (Maricq et al. 1999), (Ahlvik et al. 1997), (Williams and Swarin 1979), (Begeman and Colucci 1970). Since acceleration/deceleration are more likely to occur at intersections, emissions should show spatial dependencies along the road network. Cold-engine emissions should also show spatial dependencies, because cold starts are more likely to occur in the morning and initiate from residential population centers. As a result, Individual exposures

generated by the model, using default parameters obtained from the literature (Beyea et al. 2005), (Beyea, J., Hatch, M., et al., submitted), showed strong variation with spatial location (especially near traffic intersections).

Our estimates of historical exposure complement short-term PAH biomarker measurements carried out in the same population using PAH-DNA adducts, which are indicative of recent DNA damage (Santella 1999). Geographic modeling allows us to explore PAH exposure in the more distant past, which could be important for breast carcinogenesis. Our estimates also complement markers of PAH exposure derived from questionnaire data on diet and smoking history (http://epi.grants.cancer.gov/LIBCSP/projects/Questionnaire.html).

Although historical exposure models can generate individualized estimates of high specificity, the accuracy of the predictions may be uncertain because of reliance in some parts of the model on parameters that are known imprecisely or only by inference. Consequently, we checked our model predictions against field data (model validation), followed by optimization of parameters to improve agreement with field data (model calibration). Model validation was integrated into the original study design. As discussed in the main paper, as part of the LIBCSP study interview, interviewers collected samples from residential soils (Gammon et al. 2002a) for use in model validation and calibration. Data not specifically collected for model validation were also available for use in validation exercises, namely PAH-DNA adduct blood measurements (Gammon et al. 2002b) and PAH concentrations in extracts of residues from residential carpet dust (Gammon et al. 2002a). All validation and calibration efforts made use of the full set of data collected for both cases and controls, but with the removal of any distinguishing information on case/control status.

One of our goals with calibration was to quantify the extent to which emissions increase at intersections. Intersections are known, at least qualitatively, to be emission hot spots (Sheu et al. 1996a; Sheu et al. 1996b), (Sculley 1989), a spatial effect that is sometimes ignored in epidemiologic studies of traffic-related illness, namely those that take their exposure marker as proportional to traffic counts and/or distance to nearest major road (Brunekreef et al. 1997), (Waldron et al. 1995), (Savitz and Feingold 1989).

Data transformation.

The geographic model predictions for soil, dust, and adducts are linearly related to the emission terms, e.g., intersection emissions and cruise emissions, which would allow the use of multilinear (multiple) regression to obtain the optimized emission parameters, were it not for the fact that the distribution of the data and model predictions for individuals are log-normally distributed. Once the data and model predictions were log-transformed, non-linear regression was required. In addition, model parameters such as deposition velocity, washout rate, and photodecay rates all enter the model in a non-linear fashion. Covariates, such as BaP food index assessed from responses to a food frequency questionnaire and the number of smoked and grilled foods eaten in the most recent decade of life, were also transformed to bring them to normal form.

Soil data.

Soil samples were collected by the study interviewer in 1996-1997 to 2-cm depth via augur from the yards of randomly chosen female subjects who had lived at their current residence for at least 15 years (Gammon et al. 2002a).

The average residence time for the subjects from whom soil samples were collected was 27 years in comparison to 18 ½ years for the entire study population. Four soil samples were taken at each residence: in the center of the front and rear yards and at foundation locations by the front and rear doors. 16 PAH species including Benzo(a)Pyrene (BaP) were assayed via high-resolution GC-MS of composite samples from each residence. A subset of samples was analyzed before aggregation to determine within-yard variation (GSD = 3.0). Failure to collect soil was usually due to lack of a yard rather than weather conditions. Subjects were selected for use in our validation exercises, if they had measurements at all 4 locations at their residence (N=654) and they had provided sufficient address information to allow accurate geocoding to the street level (n = 588).

The GSD of BaP in soil, after adjustment for model deposition patterns, was 4.6, which is a measure of the between-yard variance. As mentioned above, the within-yard GSD was 3.0. Subtracting out the within-yard GSD contribution assuming lognormal distributions leaves an unexplained error term with a GSD of 2.7. Potential contributors to this residual variance include soil type, lawn care, landscaping, and building geometry. Limitations of the model could also play a role.

Two corrections were made to the soil data points to standardize them. The first accounted for a drift in calibration that was found prior to replacement of a gas chromatograph. The second correction to the soil data accounted for decay of PAH with time during freezer storage. Information was available to allow data for 500 residences to be standardized. Analysis of the soil data showed that log soil variances were fixed regardless of soil level, so chi-square for the log soil data is proportional to the standard sum of squares of differences between model and data.

Correlations of other PAHs with BaP in soil were quite high for the high-ringed compounds. For instance the following PAH/BaP Pearson correlations had r-values above 0.9: benzo(b)fluoranthene, benzo(k)flouranthene, indeno(1,2,3-cd)pyrene, benzo(ghi)perylene, and dibenz(a,h)anthracene. Acenaphthylene, fluorine, anthracene, chrysene, and benz(a)anthracene had correlation coefficients with BaP above 0.8. The remaining five light PAHs had r-values above 0.6 (naphthalene, acenaphthene, phenanthrene, fluoranthene, and pyrene). The poorer correlation for the low-ringed PAHs is not unexpected. They tend to revolatilize from surfaces (Dimashki et al. 2001), a phenomenon that we did not model. Also, they tend to be associated more with diesel emissions than with passenger vehicles, which we did not model in detail either (Marr et al. 1999), (Wingfors et al. 2001).

Fits of the soil-optimized model to soil BaP values at individual residences are shown in S-1. Even with the default model, before optimization, the confidence limits on the slope of regression line were very narrow and the p-values very small ($< 10^{-4}$), which allowed us to use the soil data for calibration. The R^2-value of the fit in the figure is 8%. (It was 3% before optimization.). A small R^2 indicates large variance, but by itself, is not a measure of the

correctness of a model (Rothman and Greenland 1998). In our case, the residuals are well behaved, meeting tests for normality and constant variance across the range of model predictions. Most importantly, the mean of the residuals do not differ significantly from zero as the model prediction changes.

As shown in the main paper, when we aggregated the data into 20-quantiles, no deviation from linearity was apparent and the R^2-value increased to 86%. Large residual variance in soil concentrations at the individual residence level is unavoidable, due to such factors as the convoluted paths material takes through the soil (Bundt et al. 2000), (Aeby et al. 2001), differences in soil type, the variation in lawn care and mowing, and the modifications of wind fields caused by different densities and shapes of buildings and surrounding trees (Thompson 1993). As long as there is no significant bias in these measurement errors, the soil data can be used for calibration. The well-behaved, linear fit to the quantile data across the full range of soil values suggests that there is no bias issue with the soil data.

PAH DNA-Adducts.

608 women had both detectable adduct levels and sufficient address information to allow accurate geocoding to the street level. The comparable number of non-detects was 248. To be sure that women lived at a location long enough for PAH-adducts to have reached equilibrium, we excluded women from our validation efforts who had lived at their current address for less than 2 full years. This reduced the number of detects to 506 and the number of non-detects to 203. An additional 21 detects were excluded because their addresses included an apartment number, indicating a possible significant discrepancy between an individual residence's true location and the centralized location of the apartment complex that defined the geocoded latitude and longitude, either vertically or horizontally. Exclusion of these subjects for the DNA-adduct comparisons, either for short duration of residence or apartment dwelling, made no difference to any conclusion reported here.

Table S-1 shows how the number of detects and non-detects for PAH-DNA adducts varies with 16-km zone along the length of Long Island. The proportion of non-detects to detects

does not differ significantly by zone (p= 0.23). Variances of log adduct data were taken as constant, based on Dickey (Dickey et al. 1997). Thus chi-square for the log soil adduct is proportional to the standard sum of squares of differences between model and data. The fit of the adduct-optimized model to the individualized adduct data is shown in Figure S-2.

Dust samples.

Dust was obtained from a random sample of homes of women, who had resided for 15 years or more at their current address (n = 320 cases and 356 controls) (Gammon et al. 2002a). Carpet dust was collected by the HVS3 vacuum from a surface area greater than 2.2 m^2 in a room occupied frequently in the year before enrollment in the study. Up to 2 g of sieved (<150 um) dust was extracted in 6% diethyl ether/ 94% n-hexane, cleaned with florisil, and analyzed by GC/MS selected ion monitoring. 556 women with dust samples provided sufficient address information to allow accurate geocoding to the street level. Two women had missing data and another 26 were excluded from analysis because their addresses included an apartment number, which implied a possible mismatch between true and geocoded location, vertically or horizontally.

As shown in Table S-2 and Figure S-3, the trend in carpet BaP/m^2 rises and then falls as one moves along 16-km zones on Long Island that mark out decreasing levels of urbanization, indicating that some source of PAH other than outside air is dominating BaP in carpets. The peak is not due to increased amounts of dust vacuumed from carpets. That quantity declines steadily (Table S-3). Therefore, the peak in BaP/m^2 is largely due to increased BaP per gram.

The spatial pattern for BaP by zone is both puzzling and interesting. Since ambient PAH has been found to account for 50% of indoor airborne PAH in a number of studies (Dubowsky et al. 1999; Sheldon et al. 1992; Sheldon et al. 1993), it is hard to see how PAH from cooking could produce a 3-fold increase in dust PAH from zones 1 to 3. As discussed in the main text, track-in of dust is another possible spatial confounder. There is only one study of which we are aware that has looked at dust track-in (Chuang et al. 1995) and that was a pilot study in Columbus, OH of 8 homes. PAH concentrations were found to be higher in entryway dust than house dust in every house studied. The concentrations were also highly correlated (Chuang et al. 1995), with a

correlation coefficient of 92%. The source of entryway (door mat) PAH is not known. Possibly, it came from street dust, which is rich in traffic PAH, or from automotive deposits in driveways or in attached garages. If so, the relationship between traffic PAH and house dust could be very complex, involving pathways that may vary from house to house and lifestyle to lifestyle.

Correlation between aggregated environmental data.

Correlations among the adduct, dust, and soil PAH measurements presented in Table 1 of the main paper were computed. The results are shown in Table S-4. Note that the individual environmental samples for adducts, dust, and soil were not necessarily collected in the same homes. Soil and dust samples had the greatest percentage of matching residences.

Carbon Monoxide data.

In general, hourly patterns of PAH and CO air concentrations are similar indoors and out (Lim et al. 1999), with r^2 coefficients ranging from 0.5 to 0.8 (Ekberg 1995; Johnson et al. 2004; Lodovici et al. 2003; Menichini et al. 1999). Figures S-4 and S-5 show the results of the multilinear fits to the CO data before and after optimization of the scale factor for intersections and distant sources (background term). Before optimization, the model fails to match the flat afternoon peak. Note that the background term has an hourly dependence. No intercept constant was allowed in the multilinear regressions. The scale factors are relative to the default model. For distant souces the CO-derived value was 5, which is identical to the factor derived from fits to soil data. The CO-derived, scale factor for intersection emissions differed by a factor of 2 from the value derived from the soil data. Because CO and PAHs are different pollutants, it would not be expected that their model parameters would necessarily agree. Nevertheless, the increased importance of emissions at intersections over the default CO-model lends additional support to the idea that intersections must be accounted for in epidemiologic analysis of traffic pollution to fully characterize the spatial distribution of exposures.

We analyzed, but did not make use of, U.S. EPA data for second CO monitor on Long Island, namely data collected for 18 months in 1973-74 at the East Farmingdale Water District in

Suffolk County (Finfer, EZ 1998, personal communication). Whereas traffic counts and other site variables are complete for the Nassau County monitoring location, we lack counts along two of the bordering roads for the Suffolk site. Presumably, the actual counts along these minor roads were low, but their absence means we lack intersection data at the corners of the four streets that surround the site. More problematic is our lack of knowledge of the actual location of the Suffolk monitor. The monitor no longer exists and two separate latitude and longitude values appear in USEPA documentation for it. In our preliminary comparisons, we chose to use the coordinates that brought the monitor closest to the location of the water district that corresponds to where staff say existing monitors of other pollutants are now located.

The fit to the CO data from the Suffolk County monitor (not shown) is not as good as the fit to the Nassau data, with the model prediction showing an early evening peak that is too strong and a morning peak that is too weak. However, we do not take the discrepancy as indicative of a need to refine the model, because of the missing intersection traffic counts and the ambiguity in monitor location.

Geocoding.

Only 10% of work addresses given at interview would fully geocode to the street level, although almost all were determinable at the city or town level. Therefore, we prepared a work exposure index imputed for each town or city for each month from 1960 to 1997. For this purpose, hypothetical workplaces were assigned along the major roads in the town or city in which a woman worked. Exposure indexes were computed for each hypothetical location in a town or city and averaged. Such an index serves at least to differentiate exposures in the more urbanized towns from the more rural villages on Long Island. Inclusion of work exposures imputed to the town or city level of the workplace worsened the fits to the adduct data. As a result, imputation was abandoned and all exposures were taken as residential exposure opportunity.

We chose two levels of accuracy for accepting geocoded addresses. The first allowed extrapolation of address number from the start of a dead-end street. For the more accurate level,

we excluded residences on dead-end streets, which allowed interpolation of address numbers between known values on either side of the geocoded location (two-sided interpolation). No difference was found in the fits to soil data when one-sided or two-sided address interpolation was used, so one-sided interpolations were accepted, which allowed exposure estimates for more women to be computed.

Background emissions

Previous traffic models have used gasoline sales data to estimate background emissions or have used a constant value as a surrogate for the cumulative contribution of distant roads (Viras et al. 1987), (Raaschou-Nielsen et al. 2000). We have attempted to directly account for traffic emissions on major roads within 80 km of our study area. However, there are more distant roads, as well as other sources of PAH emissions; therefore, it was prudent to include a background term in our model, as well. As stated in the main paper, we have tested two options for use in model calibration exercises. In the first, a constant term is added to the model prediction. For the second option, we allow background to be proportional to the exposure calculated from the more distant counties (all but Nassau, Suffolk, and Queens counties). In both approaches, we used soil or adduct data to fix the value of the unknown scale factors.

Optimized models.

Uncertain parameters in the traffic exposure model, such as particle deposition velocity, relative vehicle emission rates at intersections, and relative emissions during cold-engine conditions, were optimized by computing values on a grid within parameter space and taking the parameter values that minimized the chi-square sum of squares. Chi-square values changed slowly, indicating a well-behaved parameter space. Results are given in Table 2 of the main paper. In this supplement, we provide further details.

For the adduct and dust models, we also controlled for possible confounding variables, such as the number of smoked and grilled foods eaten in the most recent decade of life. To accomplish this, we performed a multilinear regression at each point in the model parameter

space. Let G be the prediction of exposure at a particular point in model parameter space, and let C and D be covariates transformed to normal form. To obtain a revised chi-square at each point we performed a multilinear regression to solve the following type of equation, using standard subroutines (Press et al. 1992) : $Ln(data) = a_1*Ln(G) + a_2*C + a_3*D$. We then took the output chi-square of the computation and assigned it to the grid point for the particular model, G. Thus, in effect, we use the covariates to define a transformation on the original grid of chi-square values. Since the chi-square transformations made only a small shift in the minima, we have retained the original parameter values in Table 1. All the optimized parameters for the soil model appear to have reasonable values, but it is necessary to recognize that the values obtained may have been influenced by limitations of the model and study design. For instance, although we have accurate geocoded locations *along* a street, geocoding computer programs assign a default value to distance *from* the street, not the actual value, because no such information is normally collected. It is possible that the optimization routines favored intersection emissions more than is appropriate, because the angular geometry of exposures from intersections makes them less sensitive to the perpendicular distance to the street and therefore more accurate than our estimates of cruise emissions.

In the soil model, intersections contributed 80% of the total emissions. Because the fraction of the road network where enhanced intersection emissions exist is relatively small, the fact that 80% of total emissions come from these short stretches indicates that emissions per unit length at intersections are very high.

Deposition velocity in the soil model was optimized at 0.007 m/s, ~ twice the default value. Bootstrap calculations indicate, however, that there is a broad distribution for optimized deposition velocity. Optimized rain washout and photo-decay rates were ½ and ¼ the default values, respectively. However, it should be noted that deposition, washout, and photo-decay are all plume depletion processes, and the data are not sufficient to separate out their individual contributions very precisely.

Fits of the adduct data to the warm-engine version of the model using default parameters produced p-values of the order of 0.02. However, the p-values dropped below 0.001, when we

shifted to the default, cold-engine version, indicating that the cold-engine version provides a better fit to the adduct data. Therefore, the PAH-DNA-Adduct data was used for optimization of the cold-start model.

The optimized parameters resulting from the adduct data put PAH depletion phenomena to zero, an unexpected result which may reflect compensation for model limitations, particularly the inability to capture study subject movement outside the residence. Had we been able to account for time spent away from home, such as time spent at work, we would have effectively averaged the signal over a not-insignificant area, thereby increasing the relative contribution of sources some distance from the residence.

Another parameter of interest for the cold-engine version of the model is the distance it takes for a vehicle to warm up, which is about 1-km according to the literature. The optimized value turned out to be ½-km, i.e., half as much as the default value. Although not an unreasonable travel length for warm-up to occur, this value should not necessarily be used in other studies that rely on other cold-engine models, without first considering the other length parameter optimized in the fit, namely the distance from intersections that emissions are elevated due to acceleration/deceleration effects. This length increased to ½-km, which is rather a long stretch for elevated emissions. Perhaps, these two length parameters have been jointly adjusted by the optimization algorithms to overcome simplifications in the cold-engine version of the model.

As for choice of soil-optimized model vs. adduct-optimized model, there are arguments that can be made in favor of both versions. The soil fits are cleaner, with tighter confidence intervals and larger R^2 values. Unlike the adduct-optimized model, the usefulness of the soil-optimized model does not depend on the assumed connection between DNA-adducts and cancer. Soil data retain some degree of historical deposition record. On the other hand, the adduct data implicitly account for movements inside and outside the residence. Also, the adduct data are sampling PAH within the body, which means the data are more reflective of the actual particle sizes that pass through, or metabolize within, lung tissue, allowing PAH to reach blood (Gerde and Scott 2001). In contrast, the soil PAH might include contributions from larger

particles, such as resuspended tire particles, that disperse differently than vehicle exhaust, possibly producing some degree of mismatch between the spatial pattern of particles reaching the deep lung and those depositing on soil.

Acknowledgements: CO data for the Suffolk County monitor were provided by Ed Finfer, U.S. EPA. The individual foundations and organizations that provided a supplemental grant for our work were too numerous to fit within the 50-word limit of the acknowledgement section in the main paper. We list the organizations here: Long Island Breast Cancer Coalition, Babylon Breast Cancer Coalition, West Islip Breast Cancer Coalition for L.I., Inc., Huntington Breast Cancer Action Coalition, Noreen T. Holland Breast Cancer Foundation, Inc., Breast Cancer Grassroots Organizations for a Unified Purpose, Inc.

Citations

Aeby P, Schultze U, Braichotte D, Bundt M, Moser-Boroumand F, Wydler H, et al. 2001. Fluorescence Imaging of Tracer Distributions in Soil Profiles. Environ Sci Technol 35:753-760.

Ahlvik P, Almen J, Westerholm R, Ludykar D. 1997. Impact of a block heater on regulated and some unregulated emissions from a gasoline fueled car at low ambient temperatures Technical Report 972908. Warrendale, PA: Society of Automotive Engineers.

Begeman C, Colucci J. 1970. Polynuclear aromatic hydrocarbon emissions from automotive engines Technical Report 700469. Warrendale, PA: Society of Automotive Engineers.

Beyea J, Hatch M, Stellman DH, Teitelbaum SL, Gammon MD. 2005. Development of a traffic model for predicting airborne PAH exposures since 1960 on Long Island, New York. Report to the National Cancer Institute and the National Institute of Environmental Health Sciences for work completed under USPHS Grant U01-CA/ES-66572. Lambertville, NJ 08530:Consulting in the Public Interest. Available: http://www.cipi.com/PDF/beyea2005trafficpahmodel.pdf [accessed 1 July 2005].

Brunekreef B, Janssen NA, de Hartog J, Harssema H, Knape M, van Vliet P. 1997. Air pollution from truck traffic and lung function in children living near motorways. Epidemiology 8(3):298-303.

Bundt M, Brecht A, FroideVaux P, Blaser P, Fluhler H. 2000. Impact of Preferential Flow on Radionuclide Distribution in Soil. Environ Sci Technol 34:3895-3899.

Chuang J, Callahan P, Menton R, Gordon S, Lewis R, Wilson N. 1995. Monitoring methods of PAHs and their distribution in House Dust and Track-in Soil. Environmental Science & Technology 29:494-500.

Dickey C, Santella RM, Hattis D, Tang D, Hsu Y, Cooper T, et al. 1997. Variability in PAH-DNA adduct measurements in peripheral mononuclear cells: implications for quantitative cancer risk assessment. Risk Anal 17(5):649-656.

Dickhut RM, Canuel EA, Gustafson KE, Liu K, Arzayus KM, Walker SE, et al. 2000. Automotive sources of carcinogenic polycyclic aromatic hydrocarbons associated with particulate matter in the Chesapeake Bay Region. Environ Sci Technol 34(21):4635-4640.

Dimashki M, Lim LH, Harrison RM, Harrad S. 2001. Temporal trends, temperature dependence, and relative reactivity of atmospheric polycyclic aromatic hydrocarbons. Environ Sci Technol 35(11):2264-2267.

Dubowsky SD, Wallace LA, Buckley TJ. 1999. The contribution of traffic to indoor concentrations of polycyclic aromatic hydrocarbons. J Expo Anal Environ Epidemiol 9(4):312-321.

Dunbar JC, Lin CI, Vergucht I, Wong J, Duran JL. 2001. Estimating the contributions of mobile sources of PAH to urban air using real-time PAH monitoring. Sci Total Environ 279(1-3):1-19.

Eder E. 1999. Intraindividual variations of DNA adduct levels in humans. Mutat Res 424(1-2):249-261.

Ekberg LE. 1995. Concentrations of NO2 and other traffic related contaminants in office buildings located in urban environments. Building and Environ 30(2):293-298.

Gammon MD, Neugut AI, Santella RM, Teitelbaum SL, Britton JA, Terry MB, et al. 2002a. The Long Island Breast Cancer Study Project: Description of a multi-institutional collaboration to identify environmental risk factors for breast cancer. Breast Cancer Res Treat 74(3):235-254.

Gammon MD, Santella RM, Neugut AI, Eng SM, Teitelbaum SL, Andrea Paykin A, et al. 2002b. Environmental toxins and breast cancer on Long Island. I. Polycyclic aromatic hydrocarbon (PAH)-DNA adducts. Cancer Epidemiol Biomarkers Prev 11(8).

Gerde P, Scott BR. 2001. A model for absorption of low-volatile toxicants by the airway mucosa. Inhal Toxicol 13(10):903-929.

Harkov R, Greenberg A, Darack F, Daisey JM, Lioy PJ. 1984. Summertime variations in polycyclic aromatic hydrocarbons at four sites in New Jersey. Environ Sci Technol 18:287-291.

Johnson T, Myers J, Kelly T, Wisbith A, Ollison W. 2004. A pilot study using scripted ventilation conditions to identify key factors affecting indoor pollutant concentration and air exchange rate in a residence. J Expo Anal Environ Epidemiol 14(1):1-22.

Levy JI, Houseman EA, Spengler JD, Loh P, Ryan L. 2001. Fine particulate matter and polycyclic aromatic hydrocarbon concentration patterns in Roxbury, Massachusetts: a community-based GIS analysis. Environ Health Perspect 109(4):341-347.

Lewis-Michl EL, Melius JM, Kallenbach LR, Ju CL, Talbot TO, Orr MF, et al. 1996. Breast cancer risk and residence near industry or traffic in Nassau and Suffolk, Long Island, New York. Arch Environ Health 51(4):255-265.

Lim LH, Harrison RM, Harrad S. 1999. The contribution of traffic to atmospheric concentrations of polycyclic aromatic hydrocarbons. Environ Sci Technol 33:3538-3542.

Lodovici M, Venturini M, Marini E, Grechi D, Dolara P. 2003. Polycyclic aromatic hydrocarbons air levels in Florence, Italy, and their correlation with other air pollutants. Chemosphere 50:377-382.

Maricq MM, Podsiadlik DH, Chase RE. 1999. Examination of the Size-Resolved and Transient Nature of Motor Vehicle Particle Emissions. Environ Sci Technol 33(10):1618-1626.

Marr LC, KIrchstetter TW, Harley RA, Miguel AH, Hering SV, Hammond SK. 1999. Characterization of Polycyclic Aromatic Hydrocarbons in Motor Vehicle Fuels and Exhaust Emissions. Environ Sci Technol 33(18):3091-3099.

Menichini E, Bertolaccini MA, Taggi F, Falleni F, Monfredini F. 1999. A 3-year study of relationships among atmospheric concentrations of polycyclic aromatic hydrocarbons, carbon monoxide and nitrogen oxides at an urban site. Sci Total Environ 241:27-37.

Press WH, Teukolsky SA, Vetterling WT, Flannery BP. 1992. Numerical Recipes in Fortran 77. Cambridge:Cambridge University Press.

Raaschou-Nielsen O, Hertel O, Vignati E, Berkowicz R, Jensen SS, Larsen VB, et al. 2000. An air pollution model for use in epidemiological studies: evaluation with measured levels of nitrogen dioxide and benzene. J Expo Anal Environ Epidemiol 10:4-14.

Rothman KJ, Greenland S, eds. 1998. Modern epidemiology. Philadelphia, PA:Lippincott-Raven.

Santella RM. 1999. Immunological methods for detection of carcinogen-DNA damage in humans. Cancer Epidemiol Biomarkers Prev 8(9):733-739.

Savitz DA, Feingold L. 1989. Association of childhood cancer with residential traffic density. Scand J Work Environ Health 15(5):360-363.

Sculley R. 1989. Vehicle emission rate analysis for carbon monoxide hot spot modeling. JAPCA 39:1334-1343.

Sheldon L, Clayton A, Keever J, Perritt R, Whitaker D. 1992. PTEAM: Monitoring of Phthalates and PAHS in Indoor and Outdoor Air Samples in Riverside California. final report, vol II A933-144. Sacramento, CA: California Environmental Protection Agency Air Resources Board, (CEPAARB).

Sheldon L, Whitaker D, Keever J, Clayton A, Perritt R. 1993. Phthalates and PAHs in Indoor and Outdoor Air in a Southern California Community. In: Proceedings of Indoor Air '93. Helsinki, Finland, (3)109-114.

Sheu H-L, Lee W-J, Hwang KP, Liow M-C, Wu C-C, Hsieh L-T. 1996a. Dry deposition velocities of polycyclic aromatic hydrocarbons in the ambient air of traffic intersections. J Environ Sci Health A31(9):2295-2311.

Sheu H-L, Lee W-J, Tsai J-H, Fan Y-C, Su C-C, Chao H-R. 1996b. Particle size distribution of polycyclic aromatic hydrocarbons in the ambient air of a traffic intersection. J Environ Sci Health A31(6):1293-1316.

Thompson RS. 1993. Building Amplification Factors for Sources near Buildings: A Wind Tunnel Study. Atmospheric Environment 27A(15):2313-2325.

Viras LG, Siskos PA, Stephanou E. 1987. Determination of polycyclic aromatic hydrocarbons in Athens atmosphere. Internat J Environ Anal Chem 28:71-85.

Waldron G, Pottle B, Dod J. 1995. Asthma and the motorways--one District's experience. J Public Health Med 17(1):85-89.

Williams R, Swarin S. 1979. Benzo(a)pyrene emissions from gasoline and diesel automobiles Technical Paper 790419. Warrendale, PA: Society of Automotive Engineers.

Wingfors H, Sjödin Å, Haglund P, Brorström-Lundén E. 2001. Characterisation and determination of profiles of polycyclic aromatic hydrocarbons in a traffic tunnel in Gothenburg, Sweden. Atmos Environ 35(36):6361-6369.

Zielinska B. 2004. Phase and Size Distribution of Polycyclic Aromatic Hydrocarbons in Diesel and Gasoline Vehicle Emissions. Environ Sci Technol 38:2557-2567.

Table S-1. Comparison of the number of PAH-DNA adduct detects and non-detects by zone

Zone Number[a]	Adduct detects per zone	Adduct non-detects per zone
1	136	62
2	137	59
3	88	39
4	68	19
5	30	9
6	10	0
7	8	2
8	6	4
9	2	1

a) The difference in proportions is not statistically significant by the chi-square test ($p = 0.23$).

Table S-2. Concentrations of PAH per gram of carpet dust and per square meter of carpet in 16-km geographic zones running from the most urbanized to the most rural end of Long Island.

Zone Number[a]	Carpet BaP/m^2 (geometric mean, ng/m^2)	Std error[b]	Carpet BaP/m^2 (arithmetic mean, ng/m^2)	Std error	Carpet BaP (geometric mean, ng/g)	Std error	Carpet BaP (arithmetic mean, ng/g)	Std error	Carpet data points per zone[c]
1	570	81	2600	590	1300	130	2700	340	151
2	870	130	4100	1200	1800	170	3700	470	162
3	1400	270	6500	2100	2500	440	9800	2500	98
4	1560	350	5100	1100	2600	480	6800	1300	70
5	980	420	3700	1200	1700	590	4100	950	28
6	490	470	1400	440	630	340	1290	520	9
7	1080	870	2400	1300	760	540	1400	690	6
8	310	130	370	130	660	230	740	170	4
9	NA	NA	NA	NA	NA	NA	NA	NA	0

a) 16-km zones measured from the Nassau County border eastward along the Long Island axis.

b) Applies to upper bound of standard error range. Lower bound is less.

c) Data are only included for residences that could be geocoded to the street level.

Table S-3. Concentrations of fine (< 150 μm) dust per square meter of carpet in 16-km geographic zones running from the most urbanized to the most rural end of Long Island.

Zone Number[a]	Carpet dust (geometric mean, g/m^2)	Geometric std error (GSD)[b]	Carpet dust (arithmetic mean, g/m^2)	Std error	Carpet data points per zone[c]
1	2.27	1.13	8.02	1.87	151
2	2.06	1.13	7.14	1.26	162
3	1.71	1.15	4.11	0.57	98
4	1.65	1.19	5.31	1.53	70
5	1.68	1.40	6.10	1.81	28
6	1.30	2.09	7.55	4.39	9
7	0.70	1.46	0.98	0.35	6
8	2.11	1.36	2.42	0.72	4
9	NA	NA	NA	NA	0

a) 16-km zones measured from the Nassau County border eastward along the Long Island axis.

b) Multiply and divide the previous column by the GSD to get the standard error range.

c) Data are only included for residences that could be geocoded to the street level.

Table S-4. Pearson correlation coefficients between (aggregated) environmental data collected in zones along Long Island and shown in Table 1. R^2 values in parentheses.

	PAH-DNA adducts	Soil (ng/g)	Dust (ng/m^2)
PAH-DNA adducts	1	0.77 (0.59)[a]	0.36 (0.13)[b]
Soil (ng/g)	0.77 (0.59)[a]	1	0.43 (0.18)
Dust (ng/m^2)	0.36 (0.13)[b]	0.43 (0.18)[c]	1

a) 32% of samples collected at same residence
b) 43% of samples collected at same residence
c) 80% of samples collected at same residence

Figures:

Figure S-1. Soil PAH vs. model prediction. N= 500.

Figure S-2. PAH-DNA adduct level vs. prediction of cold start model. N = 608.

Figure S-3. Carpet PAH vs. distance away from the most urbanized regions of Long Island. Geometric means by 16-km zones

Figure S-4. Fit to CO data before optimization.

Figure S-5. Fit to CO data after optimization of intersection and background scale factors.

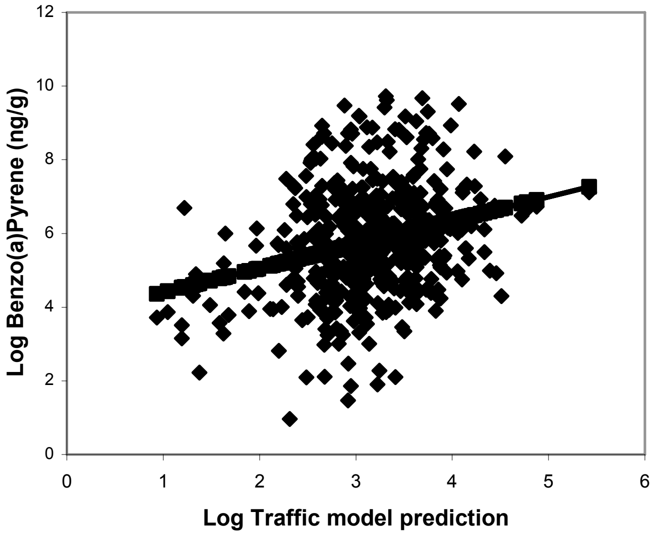

Figure S-1. Soil PAH vs. model prediction. N= 500.

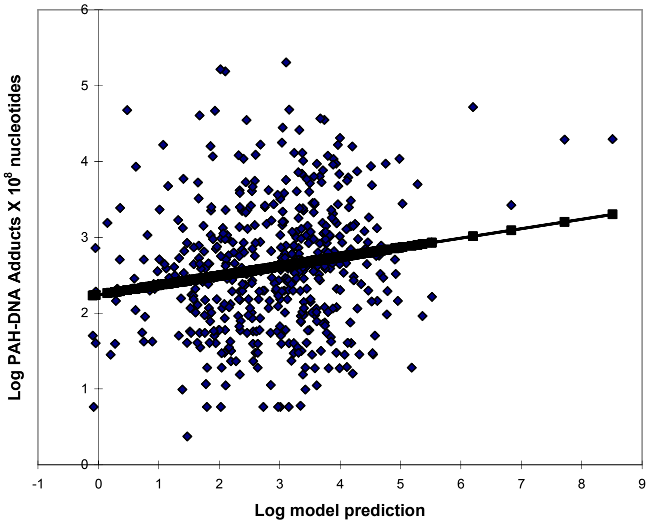

Figure S-2. PAH-DNA adduct level vs. prediction of cold start model. N = 485.

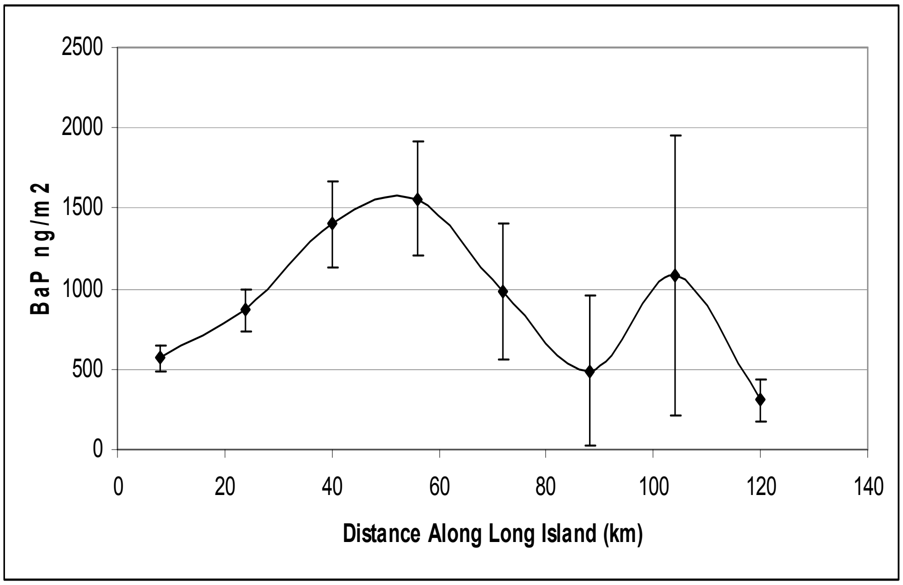

Figure S-3. Carpet PAH vs. distance away from the most urbanized regions of Long Island. Geometric means by 16-km zones.

Figures S-4 and S-5.

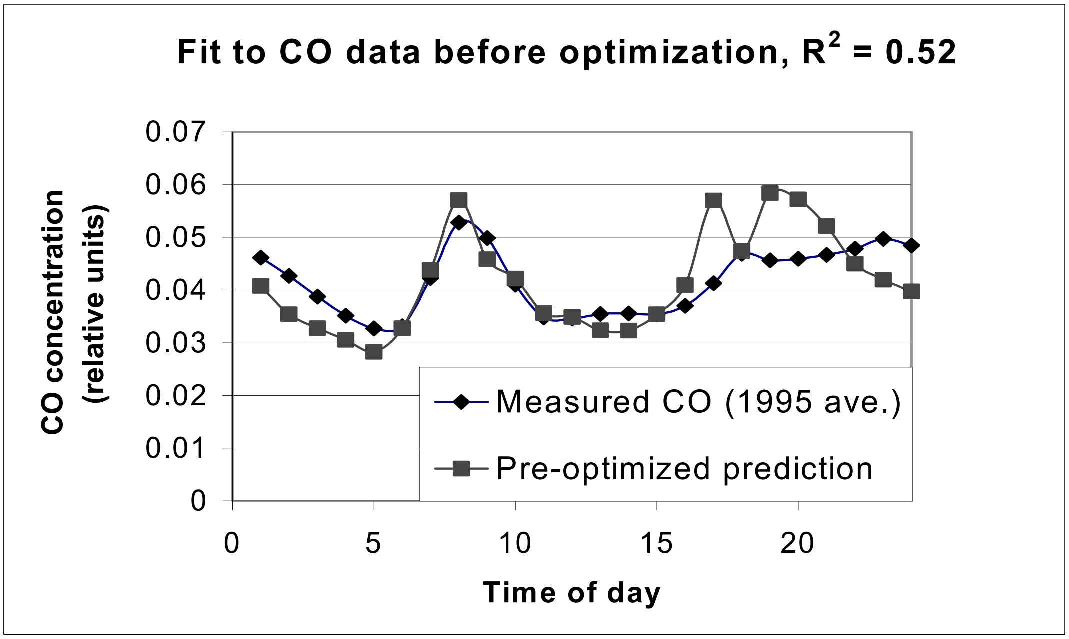

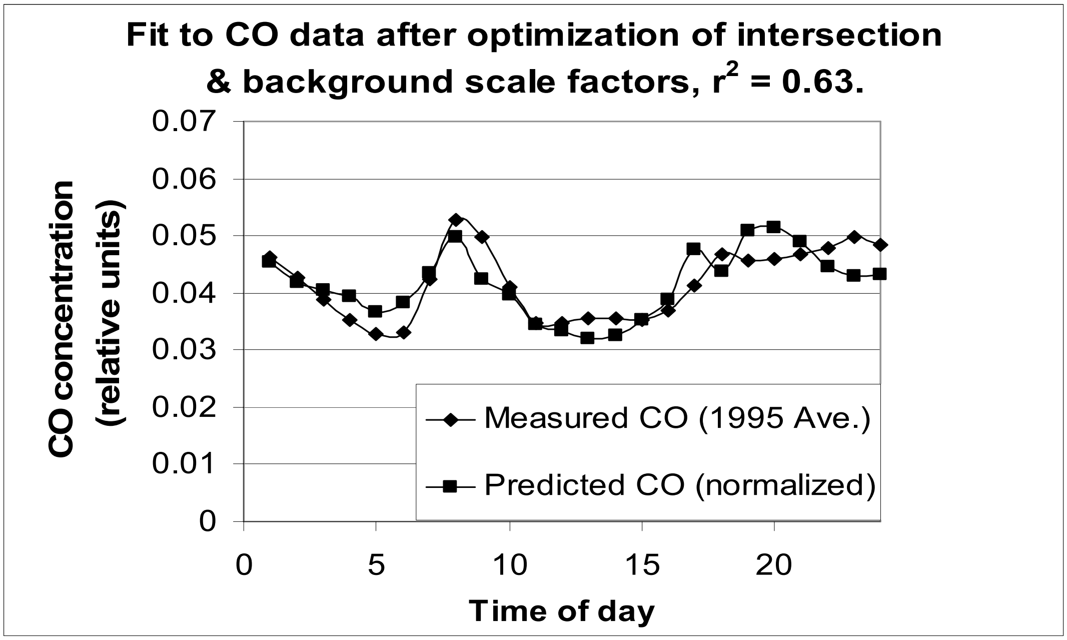

Supplement: Supplemental Figures and Tables [file ehp0114-001053s1.pdf]
